# Supplementary figures and images for: Transcriptome Analysis of Differentially Expressed Genes Associated with Salt Stress in Cowpea (Vigna unguiculata L.) during the Early Vegetative Stage
Source: Int J Mol Sci. 2023 Mar 1;24(5):4762. doi: 10.3390/ijms24054762 (PMC10002509; doi:10.3390/ijms24054762)

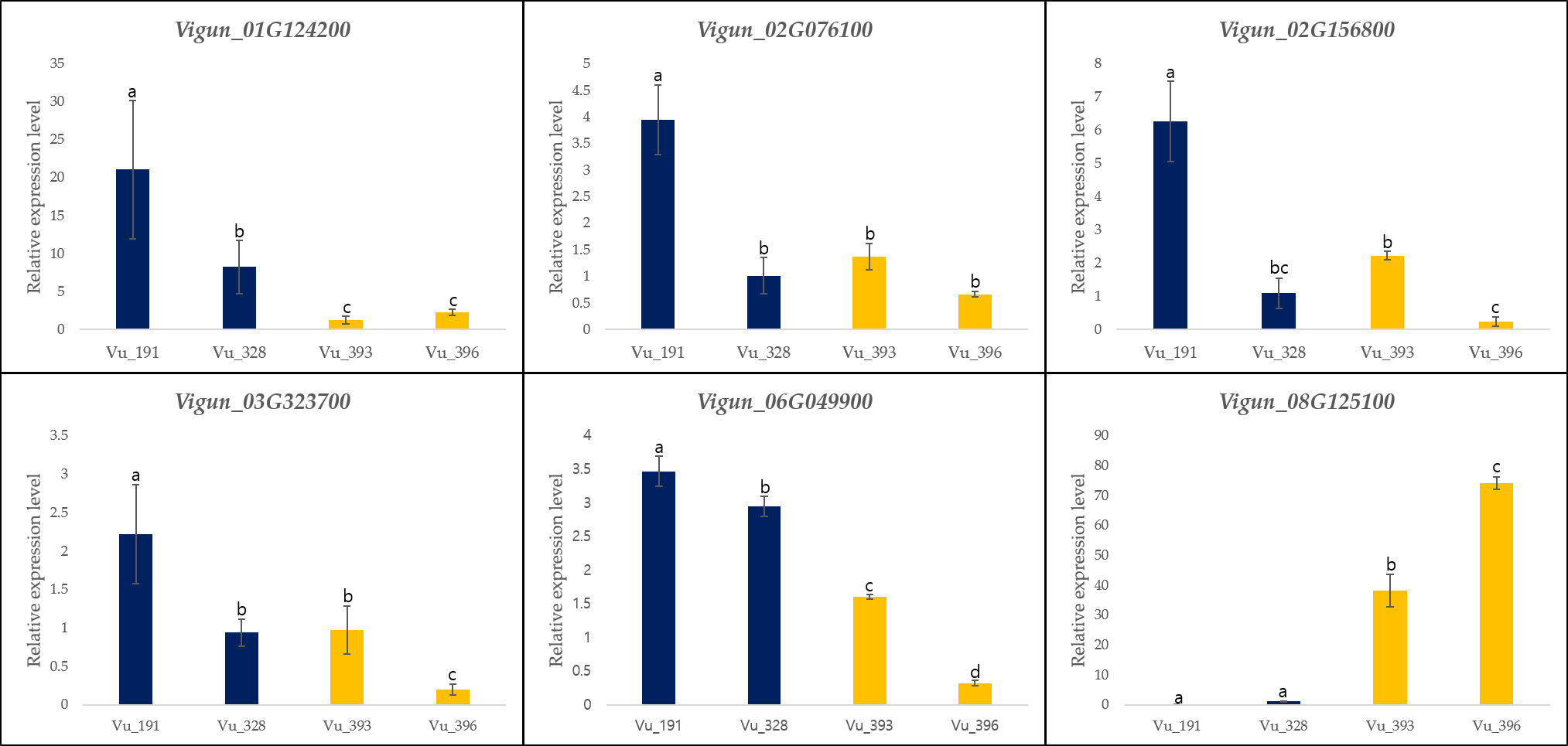

Supplement: Supplementary file 1 [file ijms-24-04762-s001.zip › Figure S1. Validation of DEGs through qRT-PCR.tif]

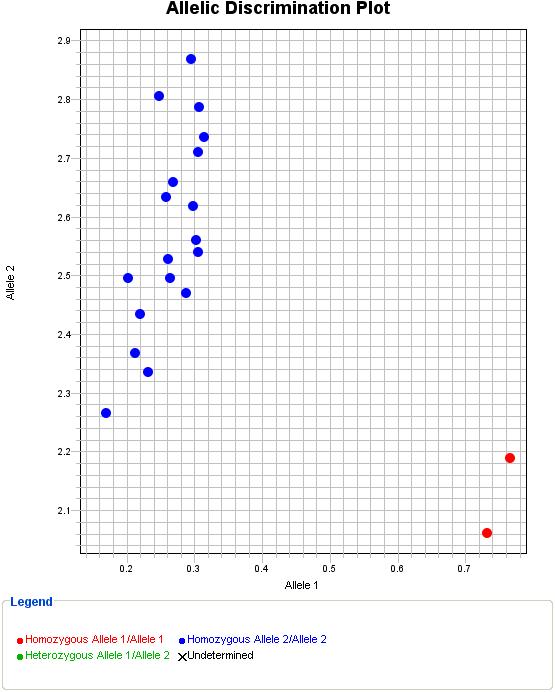

Supplement: Supplementary file 1 [file ijms-24-04762-s001.zip › Figure S2. Validation of Vigun_02G076100.jpg]

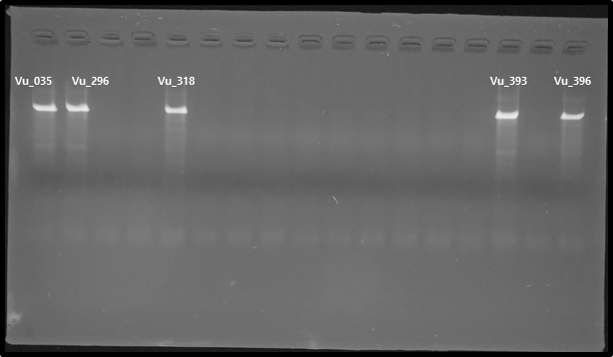

Supplement: Supplementary file 1 [file ijms-24-04762-s001.zip › Figure S3. Validation for Vigun_08G125100.tif]
